# Structural and spectral investigations of the recently synthesized chalcone (*E*)-3-mesityl-1-(naphthalen-2-yl) prop-2-en-1-one, a potential chemotherapeutic agent

**DOI:** 10.1186/s13065-015-0112-5

**Published:** 2015-06-13

**Authors:** Assem Barakat, Abdullah Mohammed Al-Majid, Saied M Soliman, Yahia Nasser Mabkhot, M Ali, Hazem A Ghabbour, Hoong-Kun Fun, Abdul Wadood

**Affiliations:** Department of Chemistry, College of Science, King Saud University, P.O. Box 2455, 11451 Riyadh, Saudi Arabia; Department of Chemistry, Faculty of Science, Alexandria University, P.O. Box 426, 21321 Alexandria, Ibrahimia Egypt; Department of Pharmaceutical Chemistry, College of Pharmacy, King Saud University, P.O. Box 2457, 11451 Riyadh, Saudi Arabia; X-Ray Crystallography Unit, School of Physics, Universiti Sains Malaysia, 11800 USM Penang, Malaysia; Department of Biochemistry, Abdul Wali Khan University Mardan, 23200 Mardan, Pakistan

**Keywords:** Aldol product, Chalcone, X-Ray, DFT compution, PAAS

## Abstract

**Background:**

Chalcones (1,3-diaryl-2-propen-1-ones, represent an important subgroup of the polyphenolic family, which have shown a wide spectrum of medical and industrial application. Due to their redundancy in plants and ease of preparation, this category of molecules has inspired considerable attention for potential therapeutic uses. They are also effective *in vivo* as anti-tumor promoting, cell proliferating inhibitors and chemo preventing agents.

**Results:**

Synthesis and molecular structure investigation of (*E*)-3-mesityl-1-(naphthalen-2-yl) prop-2-en-1-one (3) is reported. The structure of the title compound 3 is confirmed by X-ray crystallography. The optimized molecular structure of the studied compound is calculated using DFT B3LYP/6-311G (d,p) method. The calculated geometric parameters are in good agreement with the experimental data obtained from our reported X-ay structure. The calculated IR fundamental bands were assigned and compared with the experimental data. The electronic spectra of the studied compound have been calculated using the time dependant density functional theory (TD-DFT). The longest wavelength band is due to H → L (79 %) electronic transition which belongs to π-π* excitation. The ^1^H- and ^13^C-NMR chemical shifts were calculated using gauge independent atomic orbitals (GIAO) method, which showed good correlations with the experimental data (R^2^ = 0.9911–0.9965). The natural bond orbital (NBO) calculations were performed to predict the natural atomic charges at different atomic sites. The molecular electrostatic potential (MEP) was used to visualize the charge distribution on the molecule. Molecular docking results suggest that the compound might exhibit inhibitory activity against GPb and may act as potential anti-diabetic compound.

**Conclusions:**

(*E*)-3-Mesityl-1-(naphthalen-2-yl) prop-2-en-1-one single crystal is grown and characterized by single crystal X-ray diffraction, FT-IR, UV–vis, DFT and optimized geometrical parameters are close to the experimental bond lengths and angles. Molecular stability was successfully analyzed using NBO and electron delocalization is confirmed by MEP. Prediction of Activity Spectra Analysis of the title compound, predicts anti-diabetic activity with probability to have an active value of 0.348.

**Graphical Abstract:**
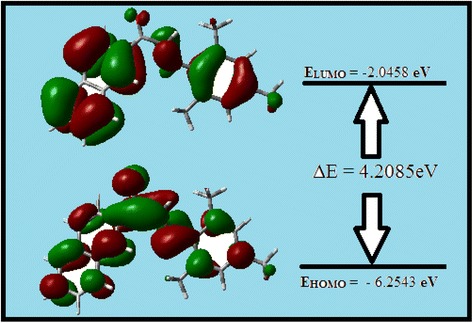
(*E*)-3-Mesityl-1-(naphthalen-2-yl) prop-2-en-1-one: a crystal structure and computational studies.

**Electronic supplementary material:**

The online version of this article (doi:10.1186/s13065-015-0112-5) contains supplementary material, which is available to authorized users.

## Background

Chalcones, constitute one of the major classes of flavonoids and isoflavonoids, with widespread distribution in fruits, vegetables, soy and tea.

Chalcones (1,3-diaryl-2-propen-1-ones), have a framework, where two aromatic rings are linked by a three carbon *α*,*β*- unsaturated carbonyl system. Contemporary studies report a generous variation of significant pharmacological activities of chalcones including anti-fungal [1–3], anti-bacterial [4], anti-tumor [5–8], and anti-inflammatory activities [9]. These activities are largely attributed to the *α*, *β*- unsaturated ketone moiety. However, the interest and development of synthetic chalcone derivatives to achieve different pharmacological activities has increased in recent years in order to establish more advanced structure-activity relationships and to generate novel compounds with diverse substituent patterns.

Chalcones is a versatile pharmacophore as compounds bearing this structural synthon possess a broad spectrum of biological activities such as anticancer potencies towards human leukemia HL-60, mouse lymphoma P388 cells [10], HeLa cell lines [11] as well as possessing antileishmanial activity [12]. In addition, several chalcones and aurones also possess an appealing pharmacological profile combining high antioxidant and lipid peroxidation activity with potent soybean LOX inhibition [13]. Julio *et al*., have prepared a series of highly functionalized chalcones. High levels of antiparasitic inhibitory activity against *Giardia lamblia* have been found within the series [14]. Anindra *et al*., have designed and studied the anticancer activity of a novel series of substituted chalcones. The preliminary anticancer activity of the tested compounds showed potent inhibitory activity towards human breast cancer cell lines [15].

The Claisen–Schmidt reaction (cross-Aldol reaction) is a condensation reaction of appropriate acetophenones derivatives with suitable aromatic aldehyde and has been playing important roles in synthetic organic chemistry [16]. Thus, the synthesis of chalcones has attracted the attention of synthetic organic/medicinal chemists. Diabetes mellitus is a principal cause of mortality and morbidity in human populations. It is a syndrome characterized by polydipsia, polyuria and hyperglycemia due to either a deficiency in the production or secretion of insulin, diminished tissue response to the actions of insulin, or both. Additionally, it causes complications to the kidneys, eyes, and nerves. It is also associated with an increased incidence of cardiovascular disease [17].

Organic molecular systems having conjugated π-systems, such as naphthalenes [18], are of great interest as potential materials for the applications related to the nonlinear optical (NLO) properties. These organic compounds are currently attracting considerable attention because of their potential applications in the optoelectronic devices of telecommunications, information storage, optical switching, signal processing [19–23] and terahertz (THz) wave generation [24]. The substituent attached to the conjugated π-system plays a vital role in terms of NLO activity. By increasing the donor-acceptor capability of the substitutions attached to the π-conjugated system, nonlinearity can be increased. The large value of the hyperpolarizability, *β*, which is the measure of the nonlinear optical activity of the molecular system, is associated with intramolecular charge transfer resulting from an electron cloud movement through a π-conjugated framework from electron donor to electron acceptor groups. The design of new systems with a high charge transfer is a key part of this, because intramolecular charge transfer between donor and acceptor will lead to a very large value for β. From this point of view, the theoretical prediction of accurate electro-optical properties for this kind of system is a very important step towards the rational design of novel nonlinear optical materials. The study of such effects involves the initial determination of static polarizabilities and hyperpolarizabilities in the gas phase.

In view of the above mentioned facts and in continuation of our interest, the structure of (*E*)-3-mesityl-1-(naphthalen-2-yl) prop-2-en-1-one **3** was unambiguously elucidated by single-crystal X-ray diffraction technique. Additionally, the DFT/B3LYP calculations have been performed to study the molecular structure characteristics of the studied compound. The electronic and spectroscopic (FTIR, UV–vis and NMR) properties of the studied compound have been predicted using the same level of theory. NBO calculations were used to calculate the natural charges at the different atomic sites. Also, molecular docking simulations for the title compound were carried out.

## Results and discussion

### Chemistry

**Scheme 1:**
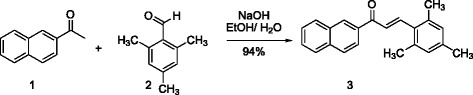
Preparation of the title compound 3

The key chalcone **3** was synthesized by a base-catalyzed Claisen Schmidt reaction between equimolar amount of 2-acetyl naphthalene and 2,4,6-trimethylbenzaldehyde in excellent yield (94 %) as illustrated in Scheme 1 [25]. Mechanistically, the reaction involves formation of a carbanion from the 2-acetyl naphthalene in the presence of base, followed by nucleophilic attack by the carbanion on the carbonyl carbon of the aldehyde and subsequent loss of water to give the chalcone.

The chemical structure of compound **3** was elucidated by its spectroscopic data including GCMS, ^1^H, ^13^C NMR, IR and single crystal X-ray structure.

### Molecular structure of compound 3

**Fig. 1:**
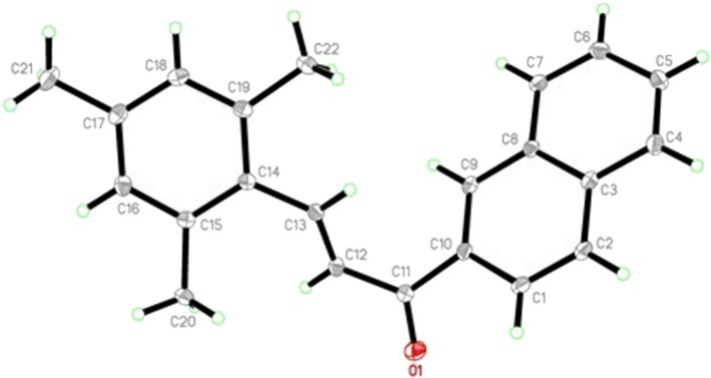
The ORTEP diagram of the final X-ray model of 3 with displacement ellipsoids drawn at 30 % probability level

**Fig. 2:**
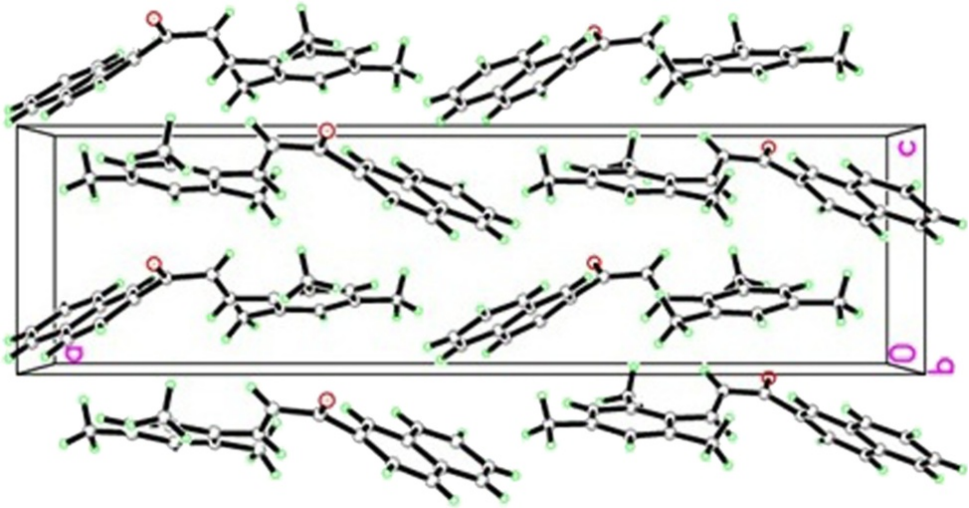
The crystal packing of 3

The asymmetric unit contains one molecule. The crystal structure of compound **3** is composed of naphthalene (C1–C10) and mesitylene (C14–C19) rings linked through prop-1-en-2-one (Fig. 1). The dihedral angle between the two ring systems is 36.24 (2) ° and the bond length between C12 and C13 is 1.342 (2) which indicates its double bond nature. The crystal packing shows that the molecules are arranged in rows along the *a*-axis and these rows are stacked by C–H · · · π interactions (see Tables 1 and 2) along the *c*-axis (Fig. 2). No significant hydrogen bonds were found. Additional file 1: Table S1. Geometric parameters (Å, °) compound **3**.

**Table 1:** The crystal and experimental data of compound 3

Crystal data	
Chemical formula	C_22_H_20_O
*M*_r_	300.38
Crystal system, space group	Orthorhombic, *Pca*2_1_
Temperature (K)	100
*a*, *b*, *c* (Å)	27.3605 (9), 7.8271 (3), 7.4710 (2)
*V* (Å^3^)	1599.94 (9)
*Z*	4
Radiation type	Mo Kα
μ (mm − 1)	0.07
Crystal size (mm)	0.67 × 0.27 × 0.16
Data collection	
Diffractometer	BrukerAPEX-II D8 Venture diffractometer
Absorption correction	Multi-scan
SADABS V2012/1 (Bruker AXS Inc.)	
Tmin, Tmax	0.94, 0.99
No. of measured, independent and observed [I > 2σ (I)] reflections	38877, 4902, 4472
Rint	0.042
Refinement	
R [F2 > 2σ (F2)], wR (F2), S	0.041, 0.107, 1.05
No. of reflections	4902
No. of parameters	211
No. of restraints	1
H-atom treatment	H-atom parameters constrained
Δρ_max_, Δρ_min_ (e Å^−3^)	0.39, −0.19

**Table 2:** Hydrogen-bond geometry (Å, °) of 3

D—H · · · A	D—H	H · · · A	D · · · A	D—H · · · A
C2—H2A · · · Cg2^**i**^	0.9300	2.92	3.7101 (17)	143
C4—H4A · · · Cg1^**i**^	0.9300	2.59	3.3807 (17)	143
C16—H16A · · · Cg1^**ii**^	0.9300	2.98	3.5506 (17)	121
C21—H21C · · · Cg2^**ii**^	0.9600	2.84	3.624 (2)	140
C22—H22B · · · Cg3^**iii**^	0.9600	2.83	3.754 (2)	162

Symmetry code: (**i**) 1-X,1-Y,-1/2 + Z. (**ii**) 1/2-X,Y,1/2 + Z. (**iii**) 1/2-X,Y,-1/2 + Z

Cg1 is the centroid of the C1–C3/C8–C10 ring, Cg2 is the centroid of the C3–C8 ring and Cg3 is the centroid of the C14–C19 ring. No significant hydrogen bonds were found

### Computational details

All the quantum chemical calculations of the studied compound were performed by applying DFT method with the B3LYP functional and 6–311G (d,p) basis set using Gaussian 03 software [26]. The input file was taken from the CIF obtained from our reported X–ray single crystal measurement. The geometry was optimized by minimizing the energies with respect to all the geometrical parameters without imposing any molecular symmetry constraints. GaussView4.1 [27] and Chemcraft [28] programs have been used to draw the structure of the optimized geometry and to visualize the MEP, HOMO and LUMO pictures. Frequency calculations at the optimized geometry were done to confirm the optimized structure to be at an energy minimum. The true energy minimum at the optimized geometry of the studied compound was confirmed by absence of any imaginary frequency modes. Vibrational mode assignments were made by visual inspection of the modes animated by using GaussView program [27]. The electronic spectra of the studied compound were calculated by the TD–DFT method. The gauge including atomic orbital (GIAO) method was used for the NMR calculations. The ^1^H and the ^13^C isotropic shielding tensors referenced to the TMS calculations were carried out at the same level of theory. The natural atomic charges were calculated using NBO method as implemented in the Gaussian 03 package [29] at the DFT/B3LYP level.

### Optimized molecular geometry

**Fig. 3:**
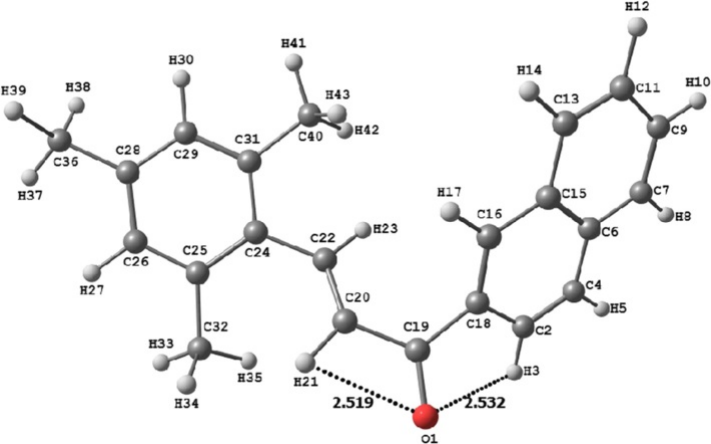
The optimized molecular structure of 3

**Fig. 4:**
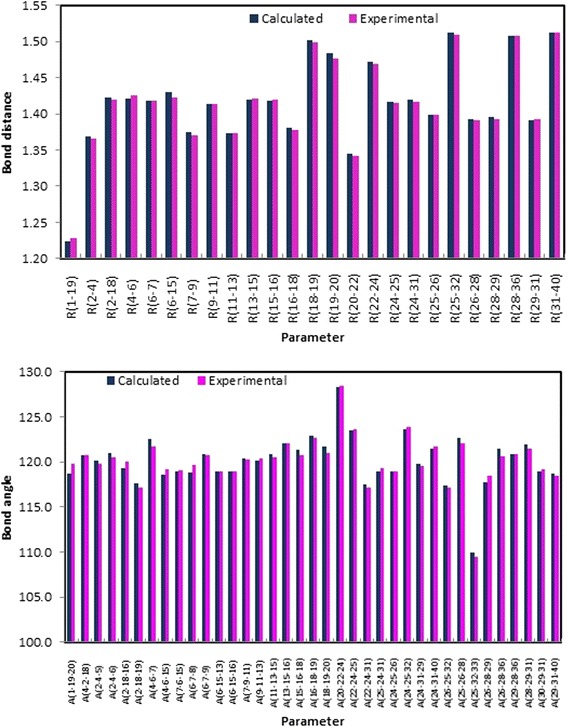
Comparison between the calculated and experimental geometric parameters (bond distances and bond angles) of 3

The optimized bond lengths and bond angles obtained for the studied compound using the B3LYP method with 6–311G (d,p) basis set are given in Table 3; while the atom numbering of the optimized structure is given in Fig. 3. The studied compound possesses C_1_ point group. The optimized geometric parameters of the studied compound are compared with those obtained from the crystallographic information file (CIF) as shown in Fig. 4. Some of these geometric parameters are overestimated while the others are underestimated. The maximum deviations of the calculated bond length and bond angle values from the experimental data are 0.005–0.007 Å (O1–Cl9 and C19–C20) and 1.058° (O1–C19–C20) respectively. These little deviations are attributed to the phase difference between the calculated and the experimental data. The root mean square deviation (RMSD), the percentage relative error and the correlation coefficient (R2) values between the experimental and calculated bond distances are found to be 0.003 Å, 0.492 % and 0.9989, respectively. Generally, the bond lengths and bond angles are predicted very well.

**Table 3:** The calculated and experimental geometric parameters of the studied compound 3 using B3LYP/6–311G (d,p) method

Parameter	Calc.	Exp	Parameter	Calc.	Exp
R (1–19)	1.223	1.228	A (4–6–7)	122.5	121.7
R (2–4)	1.369	1.366	A (4–6–15)	118.6	119.2
R (2–18)	1.422	1.420	A (7–6–15)	118.9	119.1
R (4–6)	1.421	1.425	A (6–7–8)	118.8	119.7
R (6–7)	1.418	1.418	A (6–7–9)	120.8	120.7
R (6–15)	1.430	1.423	A (6–15–13)	119.0	119.0
R (7–9)	1.375	1.371	A (6–15–16)	119.0	118.9
R (9–11)	1.414	1.413	A (7–9–11)	120.4	120.3
R (11–13)	1.374	1.374	A (9–11–13)	120.2	120.4
R (13–15)	1.419	1.421	A (11–13–15)	120.8	120.5
R (15–16)	1.418	1.420	A (13–15–16)	122.1	122.1
R (16–18)	1.380	1.378	A (15–16–18)	121.3	120.7
R (18–19)	1.502	1.499	A (16–18–19)	122.9	122.6
R (19–20)	1.484	1.477	A (18–19–20)	121.7	121.0
R (20–22)	1.345	1.342	A (20–22–24)	128.3	128.4
R (22–24)	1.472	1.469	A (22–24–25)	123.5	123.6
R (24–25)	1.416	1.415	A (22–24–31)	117.5	117.1
R (24–31)	1.420	1.416	A (25–24–31)	118.9	119.3
R (25–26)	1.398	1.398	A (24–25–26)	119.0	118.9
R (25–32)	1.512	1.510	A (24–25–32)	123.6	123.8
R (26–28)	1.392	1.391	A (24–31–29)	119.8	119.6
R (28–29)	1.396	1.392	A (24–31–40)	121.5	121.7
R (28–36)	1.508	1.508	A (26–25–32)	117.4	117.2
R (29–31)	1.391	1.393	A (25–26–28)	122.6	122.0
R (31–40)	1.512	1.513	A (25–32–33)	110.0	109.5
A (1–19–20)	118.8	119.8	A (26–28–29)	117.8	118.5
A (4–2–18)	120.7	120.7	A (26–28–36)	121.4	120.6
A (2–4–5)	120.2	119.8	A (29–28–36)	120.9	120.9
A (2–4–6)	121.0	120.5	A (28–29–31)	121.9	121.5
A (2–18–16)	119.3	120.0	A (30–29–31)	118.9	119.2
A (2–18–19)	117.6	117.2	A (29–31–40)	118.7	118.5

The calculations predicted the O1…H3 and O1…H21 intramolecular distances are 2.532 Å (exp. 2.641 Å) and 2.519 Å (exp. 2.561 Å) respectively. These results indicate the presence of weak nonconventional C–H…O intramolecular H–bonding interactions. Moreover, the C–C–C–C dihedral angles of the aromatic rings did not exceed 2.054° indicating, commonly, the planar structure of these rings. On other hand, the C16-C18-C19–O1 (33.4°), C2–C18–C19–O1 (28.2°), C31–C24–C22–C20 (33.6°) and C25–C24–C22–C20 (35.7°) dihedral angles indicated that the C19 = O1 and the C20 = C22 are not coplanar with the naphthalene and benzene rings, respectively.

### Natural atomic charge

Charge distribution has a vital role in the application of quantum chemical calculations to molecular system because atomic charges affect many properties such as dipole moment, molecular polarizability, electronic structure and acidity–basicity behaviour of compound [30]. These electronic properties have strong relations to the biological activity of compound. In this regards, the natural atomic charges (NAC) were calculated using NBO method at the DFT B3LYP/6–311G (d,p) level of theory and the results are given in Table 4. The O1 and methyl carbon (C32, C36 and C40) atoms have the highest negative natural charge values while the carbonyl carbon (C19) is the most electropositive atom in the molecule. The reason is the high electronegativity of the O-atom attached to it. All the H–atoms are electropositive. The calculated natural atomic charge values at the H-sites are in the range 0.1961–0.2212. Of the C (sp^2^)-H protons, the H3 and H21 have the highest NAC values probably due to the C-H…O interactions.

**Table 4:** The natural atomic charges calculated at the B3LYP/6–311G (d,p) method

Atom	NAC	Atom	NAC
O1	−0.5524	H23	0.2043
C2	−0.1644	C24	−0.0997
H3	0.2212	C25	0.0298
C4	−0.1730	C26	−0.2169
H5	0.2032	H27	0.1961
C6	−0.0445	C28	0.0140
C7	−0.1776	C29	−0.2200
H8	0.2024	C30	0.1960
C9	−0.1886	C31	0.0310
H10	0.2028	C32	−0.5901
C11	−0.1976	H33	0.2026
H12	0.2030	H34	0.2084
C13	−0.1710	H35	0.2171
H14	0.2006	C36	−0.5829
C15	−0.0579	H37	0.2024
C16	−0.1576	H38	0.2055
H17	0.2087	H39	0.2096
C18	−0.1253	C40	−0.5897
C19	0.5286	H41	0.2047
C20	−0.2669	H42	0.2083
H21	0.2114	H43	0.2063
C22	−0.1419		

**Fig. 5:**
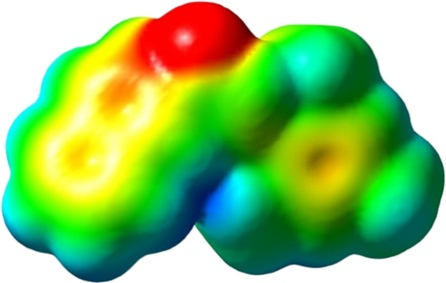
Molecular electrostatic potentials (MEP) mapped on the electron density surface calculated by the DFT/B3LYP

Molecular electrostatic potential maps are very useful three dimensional diagrams which are used to visualize the charge distributions and charge related properties of molecules. Also, MEP picture has been used to predict the reactive sites for electrophilic and nucleophilic attack, and in studies of biological recognition and hydrogen bonding interactions [31, 32]. The MEP of the studied compound calculated using B3LYP method with 6–311G (d,p) basis set is shown in Fig. 5. It can be seen from the MEP figure; negative regions (red) are mainly localized over the carbonyl oxygen atom while the positive regions (blue) are distributed over the H-atoms.

### Frontier molecular orbitals

**Fig. 6:**
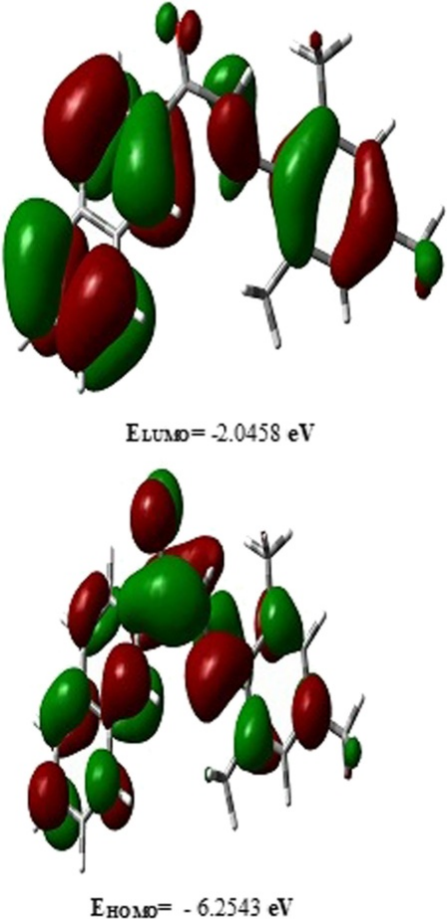
The ground state isodensity surface plots for the frontier molecular orbitals

The electron densities of the frontier molecular orbitals (FMOs) were used for predicting the most reactive position in π–electron systems and to explaine several types of reactions in conjugated system [33]. The properties of these FMOs like energy are very useful for physicists and chemists. The energy values of the lowest unoccupied molecular orbital (E_LUMO_) and the highest occupied molecular orbital (E_HOMO_) and their energy gap (ΔE) reflect the chemical reactivity of the molecule. Recently the energy gap between HOMO and LUMO has been used to prove the bioactivity from intramolecular charge transfer (ICT) [34, 35]. The E_HOMO_, E_LUMO_ and ΔE values for the studied compound were calculated by B3LYP/6–311G (d,p) method. The HOMO and LUMO pictures are shown in Fig. 6. It is found that the HOMO and LUMO levels are distributed mainly over the ring (π–system). The E_HOMO_ and E_LUMO_ are calculated to be - 6.2543 eV and −2.0458 eV respectively. The HOMO–LUMO energy gap (ΔE) represents the lowest energy electronic transition which mainly belongs to π– π* excitation (4.2085 eV).

**Fig. 7:**
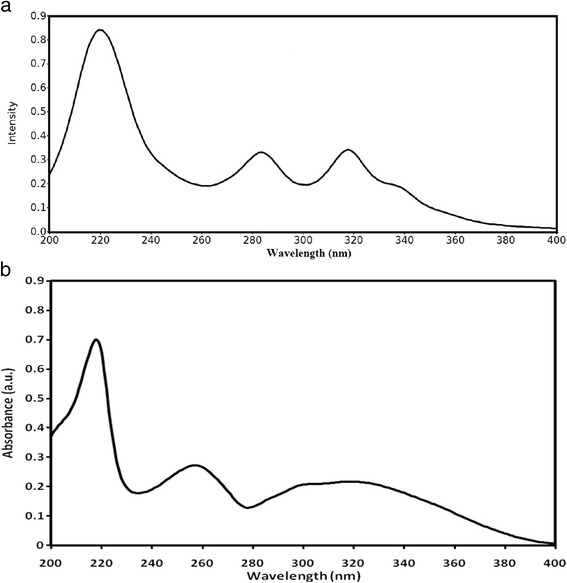
The calculated (**a**) and experimental (**b**) electronic spectra of 3

The more accurate electronic transitions were calculated using the time–dependant density functional theory (TD–DFT). The twenty spin allowed singlet-singlet electronic transitions using the TD–DFT method are given in Additional file 1: Table S2. The experimental and calculated electronic spectra are shown in Fig. 7. The studied compound showed four electronic transition bands at 216, 258, 302 and 321 nm. In agreement with the experimental data the calculations predicted these transition bands at 215.4 nm (f = 0.3187), 283.8 nm (f = 0.2028), 317.7 nm (f = 0.2765) and 338.0 nm (f = 0.1023). The longest wavelength transition band is due to H → L (79 %) excitation. The shortest wavelength band showed the highest intensity both experimentally and theoretically. The presence of some deviations between the calculated and the experimental data is attributed to the medium effect.

### Nonlinear optical properties

Nonlinear optical materials were used as key materials for photonic communications which use light instead of electron for data transmission. With the development of laser technology, nonlinear optical materials have been extensively applied to industry, medicine and research [36, 37]. Several organic materials were used for such applications. These organic compounds were characterized by their high polarizability (α_0_) and low HOMO–LUMO gap (ΔE). The α_0_ and ΔE values of the studied compound are calculated to be 265.6 Bohr^3^ and 4.2085 eV respectively. The polarizability of the studied compound is about 10 times higher than urea.

The large value of the hyperpolarizability, β is associated with intramolecular charge transfer resulting from an electron cloud movement through a π-conjugated framework. In this paper, the studied molecule was divided into two parts to evaluate the charge distribution, Part A for naphthalene ring and Part B is for the mesityl propenone moiety (Fig. 3). The total NAC value at part A is negative (−0.0156). It is obvious that Part A serves as electron acceptor, while Part B is positively charged (+0.0156) and serve as electron donor. In the studied molecule, there is significant intramolecular charge transfer (ICT) from the naphthalene ring (part A) as electron donor to part B as electron acceptor. Such ICT is responsible for the high hyperpolarizability of the studied system.

Also the studied compound has lower energy gap (ΔE) compared to urea. Based on these calculations, the studied molecule is considered as better NLO material than urea which is used as reference molecule for comparison of the NLO activity [38].

### NMR spectra

**Fig. 8:**
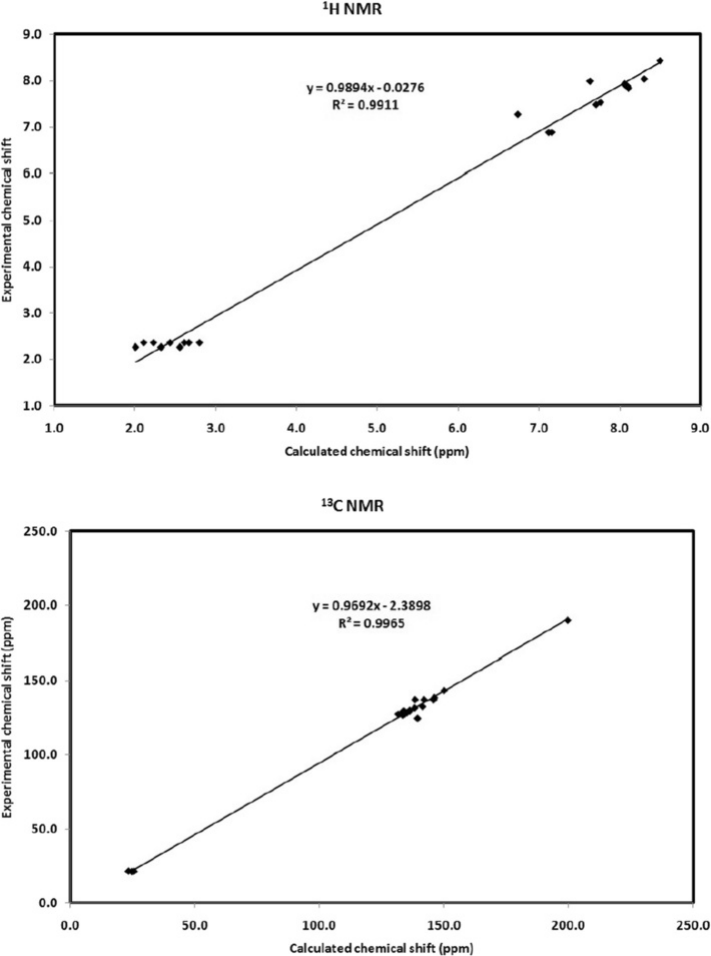
The correlation graphs between calculated and experimental ^1^H-NMR and ^13^C-NMR chemical shifts of the 3

The isotropic magnetic shielding (IMS) values calculated using the GIAO approach at the 6–311G (d,p) level are used to predict the ^13^C and ^1^H chemical shifts (*δ*_calc_) for the studied compound and the results are correlated to the experimental NMR data (*δ*_exp_) in CDCl_3_ solvent. The experimental and theoretical values for ^1^H- and ^13^C-NMR chemical shifts of the studied compound are given in Additional file 1: Table S3. According to these results, the calculated chemical shifts are in compliance with the experimental findings. As shown in Fig. 8, the agreement between the experimental and the calculated carbon–13 (R^2^ = 0.9965) and proton (R^2^ = R^2^ = 0.9911) chemical shifts are good.

### Analysis of the vibrational spectra

**Fig. 9:**
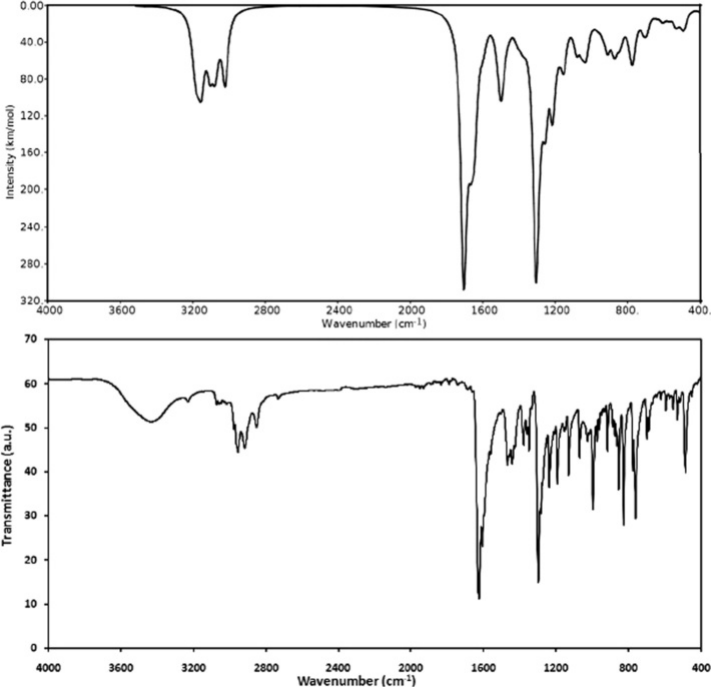
The experimental (lower) and calculated (upper) infrared spectra of the 3

**Fig. 10:**
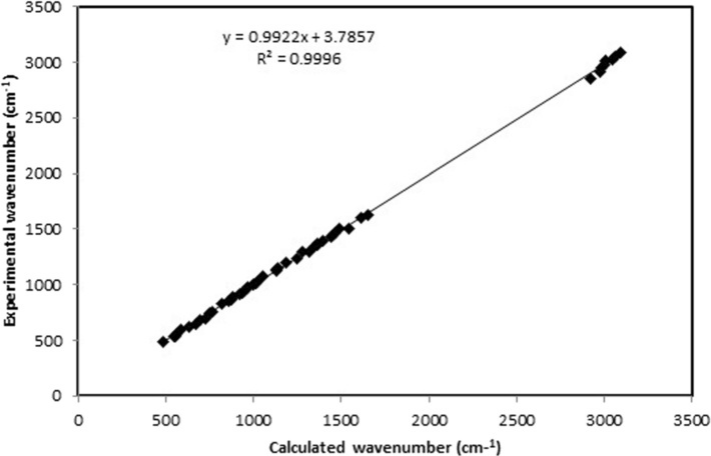
Correlation graph between the calculated and experimental vibrational frequencies of the studied compound

The infrared vibrational frequencies and intensities of the titled compound were calculated using the DFT B3LYP/6–311G (d,p) method and the results are given in Additional file 1: Table S4. The experimental and predicted IR spectra of the studied compound are given in Fig. 9. Selected calculated and experimental vibrational frequencies along with their assignments are presented in Table 5. A correlation graph between the calculated and the experimental vibrational frequencies is shown in Fig. 10. As can be seen from this figure, good correlation is obtained between the calculated and the experimental vibrational frequencies with high correlation coefficient (R^2^ = 0.9996).

**Table 5:** The calculated and experimental wavenumbers of the studied compound 3

Assignment	Calculated	Experimental
υ _(CH, aromatic)_	3096–3046	3090–3030
υ _(=C20H21)_	3092	3090
υ _(=C22H23)_	3053	
υ _(CHasym, CH3)_	3011–2971	3016–2919
υ _(CHsym, CH3)_	2928–2922	2852
υ _(C=O)_	1649	1626
υ_C20=C22_	1614	1604
υ_C=C (aromatic)_	1611–1489	1546–1500
δ_CH in-plane methyl_	1466–1441	1469–1433
δ_CH out-of-plane methyl_	1419–1396	1391
δ_methyl umbrella_	1373–1367	1365
δ_CH aromatic in plane_	1362–1333, 1265–1116, 1008	1353, 1345, 1237–1130
δ _(=C-H in plane)_	1318, 1278	1299, 1290
δ_CH aromatic out-of-plane_	967, 960, 937, 904–816, 765, 748–691	975, 960, 885–827, 760, 745–680
δ _(=C-H out-of-plane)_	995, 883	996, 885
δ_methyl rocking_	1049–1014	1070–1015

*υ* streching, *δ* bending

The studied compound has three types of C-H bonds; 9 aromatic, 9 aliphatic and two vinylene groups. The aromatic stretching bands of the studied compound are calculated at 3096–3046 cm^−1^ (except 3092 cm^−1^) [39]. The seven υ_C-H_ modes of the naphthalene ring are calculated at 3096, 3084, 3079, 3072, 3061, 3059 and 3055 cm^−1^ [39] while the two bands calculated at 3048 and 3046 cm^−1^ are assigned to the symmetric and asymmetric C-H stretching vibration of the benzene ring, respectively. Both *cis* and *trans* dialkyl substituted ethylene (RCH = CHR) have a C-H stretches in the range 3020–2995 cm^−1^ [40]. The two υ_C-H_ modes of the vinylene group are calculated at 3092 and 3053 cm^−1^. The high frequency values of these stretching modes probably due to the presence of nonalkane substituents attached to the vinylene group [40].

The aromatic ring C-H in-plane bending vibrations are calculated at 1362–1333, 1265–1116 and 1008 cm^−1^ while the C-H out-of-plane bending vibrations are calculated at 967, 960, 937, 904–816, 765 and 748–691 cm^−1^. The visual inspection of the vibrational modes showed that the C-H in-plane bending vibrations of the vinylene group are calculated at 1318 and 1278 cm^−1^ (exp. 1299 and 1290 cm^−1^) while the out-of-plane bending modes are calculated at 995 and 883 cm^−1^ (exp. 996 and 885 cm^−1^).

According to Pulay *et al*. [41], the methyl (CH_3_) group has five types of vibrational frequencies namely: symmetric stretch, asymmetric stretch, symmetric deformation, asymmetric deformation and rocking. The studied molecule has three methyl groups; the 9 stretching modes are calculated in the range 3011–2971 cm^−1^ (exp. 3016–2919 cm^−1^) and 2928–2922 (exp. 2852 cm^−1^) for the asymmetric and symmetric stretching vibrations respectively [42]. The asymmetric stretch is usually at higher frequency than the symmetric one. The asymmetric and symmetric bending vibrations of methyl groups are predicted in the region 1466–1441 cm^−1^ and 1419–1396 cm^−1^, respectively [43,434]. The umbrella modes were calculated in the range 1373–1367 cm^−1^. The rocking vibrations of the CH_3_ group appear as mixed vibrations and usually appear in the region 1170–1100 cm^−1^ [43, 44]. The CH_3_ rocking modes, which are coupled with other vibration modes, are predicted in the frequency region of 1049–1014 cm^−1^ (exp. 1049–1014 cm^−1^). Moreover, the studied compound has one carbonyl group (C = O). The carbonyl stretching vibrations generally appear in the region 1750–1600 cm^−1^. In the present case, the absorption band observed at 1626 cm^−1^ (cal. 1649 cm^−1^) are assigned to the conjugated C = O group. Also, the υ_C20=C22_ and the aromatic ring C–C vibrational frequencies are calculated at 1614 cm^−1^ (exp. 1604 cm^−1^) and 1611–1489 cm^−1^ (exp. 1546–1500 cm^−1^) respectively. These results are in agreement with the literature [45].

### Thermogravimetric Analysis (TGA)

**Fig. 11:**
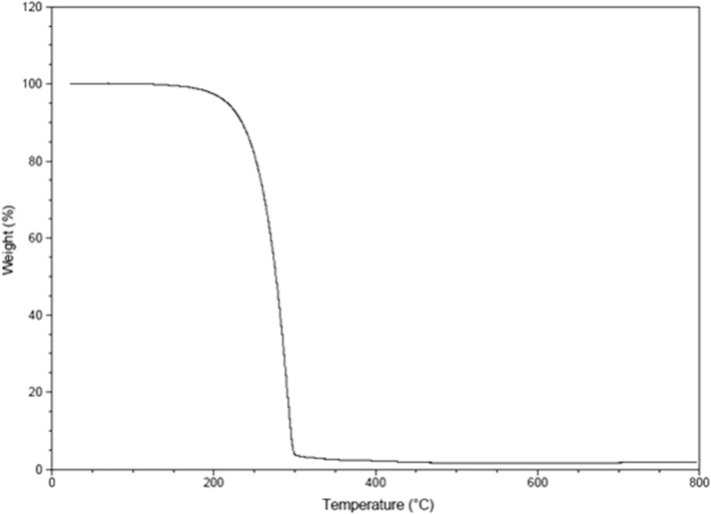
The TGA curve of 3

The TGA of the studied compound is performed over the temperature range 25–800 °C under flowing nitrogen atmosphere and the result is shown in Fig. 11. The TGA data showed that the studied compound is thermally stable up to 205 °C then undergo either sublimation or decomposition into volatile products in a fast step leaving almost 0 % residue.

### Molecular docking simulation

**Fig. 12:**
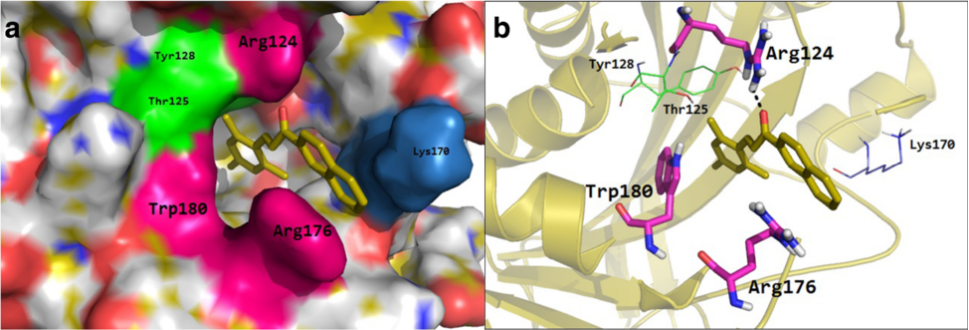
**a** The title compound 3 was fit well in the cavity of histone acetyltransferase enzyme. **b** Docking conformation of title compound 3 (generated by MOE docking software) properly accommodated into the binding cavity of histone acetyltransferase enzyme and developed hydrogen bond and two arene-cation and arene-arene interactions with active site residue Arg176, Arg124 and Trp180. histone acetyltransferase

PASS (Prediction of Activity Spectra) is an online tool [46] which predicts almost 900 types of activities based on the structure of a compound. PASS analysis (Additional file 1: Table S5) of the title compound **3** predicts anti-diabetic activity (human histone acetyltransferase) with Pa (probability to be active) value of 0.348. To evaluate the inhibitory nature of the title compound against human histone acetyltransferase, molecular docking simulations were carried out. Molecular docking is an efficient method to get an insight into ligand-receptor interactions. Molecular docking studies were performed using Molecular Operating Environment (MOE) software (www.chemcomp.com). The 3D crystal structure of human histone acetyltransferase was downloaded from Protein Data Bank (PDB ID: 4PZS) [47]. Before docking experiment, the title compound **3** was prepared for docking by minimizing its energy at B3LYP functional and 6–311G (d,p) basis set using Gaussian 03 software. Partial charges were calculated by Gasteiger method. Most macromolecular crystal structures contain little or no hydrogen coordinate data due to limited resolution and thus protonation was done prior to docking using Protonate 3D tools implemented in MOE. Protonation was followed by energy minimization up to 0.05 Gradient using Amber99 force field. The docking protocol predicted the same conformation as was present in the crystal structure with RMSD value close to the allowed range [48] and surrounded by the same active site residues of the enzyme. Amongst the generated docking conformations the top-ranked conformation was visualized for ligand-enzyme interaction using PyMol. Analysis of the docking results showed that the synthesized compound **3** fit well within the active site of histone acetyltransferase enzyme (Fig. 12a). From the docking conformation it was observed that both the naphthalene, mesitylene moiety as well as the carbonyl oxygen of 2-methylprop-1-ene moiety of the compound **3** interact with important active site residues of the enzyme, e.g., Arg124, Arg176 and Trp180. Arg176 was found in making two arene-cation interactions with both the phenyl ring of naphthalene moiety, Arg124 was involved in interaction with carbonyl oxygen of 2-methylprop-1-ene moiety and Trp180 was found in sharing its π-electron with the phenyl ring of the compound **3** and making π- π interaction by which they stabilized the structure (Fig. 12b). The presence of one H-bond, one arene-arene and two arene-cation interactions confirm that the inhibitor may be specific to this site. These preliminary results suggest that the compound **3** might exhibit inhibitory activity against histone acetyltransferase enzyme and may act as potential anti-cancer compound. However, further biological tests should be done to validate the computational predictions.

## Experimental section

### General

All chemicals were purchased from Sigma-Aldrich, Fluka etc., and were used without further purification, unless otherwise stated. All melting points were measured on a Gallenkamp melting point apparatus in open glass capillaries and are uncorrected. IR Spectra were measured as KBr pellets on a Nicolet 6700 FT-IR spectrophotometer. The NMR spectra were recorded on a Varian Mercury Jeol-400 NMR spectrometer. ^1^H-NMR (400 MHz), and ^13^C-NMR (100 MHz) were run in deuterated chloroform (CDCl_3_). Chemical shifts (*δ*) are referred in terms of *ppm* and *J* -coupling constants are given in *Hz*. Mass spectra were recorded on a Jeol JMS-600 H. Elemental analysis was carried out on Elmer 2400 Elemental Analyzer in CHN mode. The thermal analysis of the studied compound has been carried out using TGA Q500 V20.10. The wt% loss was measured from ambient temperature to 800 °C.

### X-ray crystallography

Slow evaporation of ethanol solution of pure compound **3** yielded colorless crystals. A crystal of dimensions, 0.67 × 0.27 × 0.16 mm was selected for X-ray diffraction analysis. Data were collected on a Bruker APEX-II D8 Venture area diffractometer, equipped with graphite monochromatic Mo K*α* radiation at 100 °K. Cell refinement and data reduction were carried out by Bruker SAINT. SHELXS-97 [49, 50] was used to solve structure. The final refinement was carried out by full-matrix least-squares techniques with anisotropic thermal data for non-hydrogen atoms on *F*^2^. All the hydrogen atoms were placed in calculated positions (Tables 1, 2 and 3). The asymmetric unit of the crystal structure is shown in Fig. 1 and the crystal packing is shown in Fig. 2.

The structure of **3** was confirmed by X-ray crystal structure analysis (Bruker AXS GmbH). CCDC- 1026251 contains the supplementary crystallographic data for this compound. These data can be obtained free of charge from the Cambridge Crystallographic Data Centre via www.ccdc.cam.ac.uk/data_request/cif.

### Preparation of (*E*)-3-mesityl-1-(naphthalen-2-yl) prop-2-en-1-one (3)

A mixture of 1-(naphthalen-2-yl) ethanone 1 (1.5 mmol, 255.3 mg), 2,4,6-trimethylbenzaldehyde **2** (1.5 mmol, 222.3 mg) in 10 mL EtOH. NaOH (2.0 mmol, 80 mg) was dissolved in a mixture of EtOH:H_2_O (1:1) in 10 mL and was added dropwise over 5mins, the reaction mixture was stirred at room temperature for 24 h until TLC showed complete disappearance of the reactants. The product was precipitated and filtered off washed with 20 mL water, dried and recrystallized from EtOH to afford the pure product **3**. mp. 88 °C; IR (*ν*_max_) (KBr)/cm^−1^ 3441, 2954, 2919, 2852, 1626, 1468, 1441, 1298; ^1^H-NMR (400 MHz; CDCl_3_): *δ* 2.26 (s, 3H, C**H**_3_); 2.37 (s, 6H, 2xC**H**_3_); 6.89 (s, 2H, Ph-**H**); 7.28 (d, 1H, *J* = 16.1Hz, CH = C**H**CO); 7.49 (td, 1H, *J* = 8.0 Hz&1.5Hz, Ar-**H**); 7.54 (td, 1H, *J* = 8.0 Hz&1.5Hz, Ar-**H**); 7.84 (d, 1H, *J* = 8.0 Hz, Ar-**H**); 7.89 (d, 1H, *J* = 8.8 Hz, Ar-**H**); 7.92 (d, 1H, *J* = 8.1 Hz, Ar-**H**); 7.99 (d, 1H, *J* = 16.1 Hz, C**H** = CHCO), 8.05 (dd, 1H, *J* = 8.8 Hz & 2.2Hz, Ar-**H**); 8.43 (s, 1H, Ar-**H**); ^13^C-NMR (100 MHz; CDCl_3_): 20.9, 21..2, 21.3, 124.5, 126.6, 127.6, 127.9, 128.2, 128.4, 129.2, 129.3, 129.4, 130.0, 131.6, 132.5, 135.5, 137.1, 138.5, 143.3, 190.3; MS *m*/*z* (%):300.39 [M+, 98 %]; Anal. calcd. for C_22_H_20_O: C, 87.96; H, 6.71; Found: C, 87.99; H, 6.73.

## Conclusions

The synthesis and characterization of (*E*)-3-mesityl-1-(naphthalen-2-yl) prop-2-en-1-one **3** is reported. The TGA analysis showed high thermal stability of studied compound up to 205 °C. The molecular structure of the studied compound has been optimized using the DFT/B3LYP method and 6-311G (d,p) basis set. The calculated bond distances and bond angles showed good agreement with our reported X-ray crystal structure. The molecular electrostatic potential picture of the studied compound has been calculated using the same level of theory. The MEP results showed that the carbonyl oxygen (O5) is the most electronegative and the H-atoms are the most electropositive sites. The α_0_ and HOMO-LUMO energy gap (ΔE) values indicated that the studied molecule is considered as a better NLO material than urea by 10 times. The calculated electronic spectra using the TD–DFT method showed four electronic transition bands at 215.4 nm (f = 0.3187), 283.8 nm (f = 0.2028), 317.7 nm (f = 0.2765) and 338.0 nm (f = 0.1023). The GIAO ^1^H- and ^13^C-NMR chemical shift values correlated well with the experimental data (R^2^ = 0.9911-0.9965). The IR vibrational frequencies are calculated and the fundamental bands were assigned and compared with the experimental data. Further studies towards the biological activity are in progress.

## Additional file

Additional file 1: Figure S1.
^1^H-NMR of the synthesized compound **3**. **Figure S2.** ^13^C-NMR of the synthesized compound **3. Table S1.** Geometric parameters (Å, °) compound **3**. **Table S2.** The calculated electronic transitions using TD-DFT method. **Table S3.** The calculated chemical shifts δ (ppm) of the studied compound using GIAO method. **Table S4.** The calculated unscaled and scaled infrared vibrational frequencies of the studied compound. **Table S5.** PASS prediction of the compound, Pa represents probability to be active and Pi represents probability to be inactive.
