# Supplementary material for: Structural and spectral investigations of the recently synthesized chalcone (E)-3-mesityl-1-(naphthalen-2-yl) prop-2-en-1-one, a potential chemotherapeutic agent
Source: Chem Cent J. 2015 Jun 13;9:35. doi: 10.1186/s13065-015-0112-5 (PMC4477317; doi:10.1186/s13065-015-0112-5)
Supplement: Additional file 1: Figure S1. — 1H-NMR of the synthesized compound 3. Figure S2. 13C-NMR of the synthesized compound 3. Table S1. Geometric parameters (Å, °) compound 3. Table S2. The calculated electronic transitions using TD-DFT method. Table S3. The calculated chemical shifts δ (ppm) of the studied compound using GIAO method. Table S4. The calculated unscaled and scaled infrared vibrational frequencies of the studied compound. Table S5. PASS prediction of the compound, Pa represents probability to be active and Pi represents probability to be inactive. [file 13065_2015_112_MOESM1_ESM.docx]

# Structural and spectral investigations of the recently synthesized chalcone (*E*)-3-mesityl-1-(naphthalen-2-yl)prop-2-en-1-one, a potential chemotherapeutic agent

AssemBarakat, ^1,2,^*^,§^ Abdullah Mohammed Al-Majid,^1,^* Saied M. Soliman,^2,^* Yahia Nasser Mabkhot,^1,^* M. Ali, ^1,^* Hazem A. Ghabbour,^3,^* Hoong-Kun Fun^3,4,^* and Abdul Wadood^5,^*

^1^Department of Chemistry, College of Science, King Saud University, P. O. Box 2455,
Riyadh 11451, Saudi Arabia

^2^Department of Chemistry, Faculty of Science, Alexandria University, P.O. Box 426, Ibrahimia, Alexandria 21321, Egypt.

^3^Department of Pharmaceutical Chemistry, College of Pharmacy, King Saud University,

P.O. Box 2457, Riyadh 11451, Saudi Arabia

^4^X-Ray Crystallography Unit, School of Physics, Universiti Sains Malaysia, Penang, 11800 Malaysia.

^5^Department of Biochemistry, Abdul Wali Khan University Mardan, Mardan-23200, Pakistan.

*These authors contributed equally to this work

^§^Corresponding author

Email addresses:

AB: [ambarakat@ksu.edu.sa](mailto:ambarakat@ksu.edu.sa)

AMA: [amajid@ksu.edu.sa](mailto:amajid@ksu.edu.sa)

SMS: saied1soliman@yahoo.com

YNM: [yahia@ksu.edu.sa](mailto:yahia@ksu.edu.sa)

MA: [mohamedali.eg25@gmail.com](mailto:mohamedali.eg25@gmail.com)

HAG: ghabbourh@yahoo.com

HKF: [hfun.c@ksu.edu.sa](mailto:hfun.c@ksu.edu.sa)

AW: [awadood@awkum.edu.pk](mailto:awadood@awkum.edu.pk)

^*^Corresponding author: Dr Assem Barakat, Department of Chemistry, College of Science, King Saud University, P. O. Box 2455, Riyadh 11451, Saudi Arabia; +966-11467-5884 (A.B.); Fax: +966-11467-5992; E-Mail: ambarakat@ksu.edu.sa

SUPPLEMENTARY DATA

**
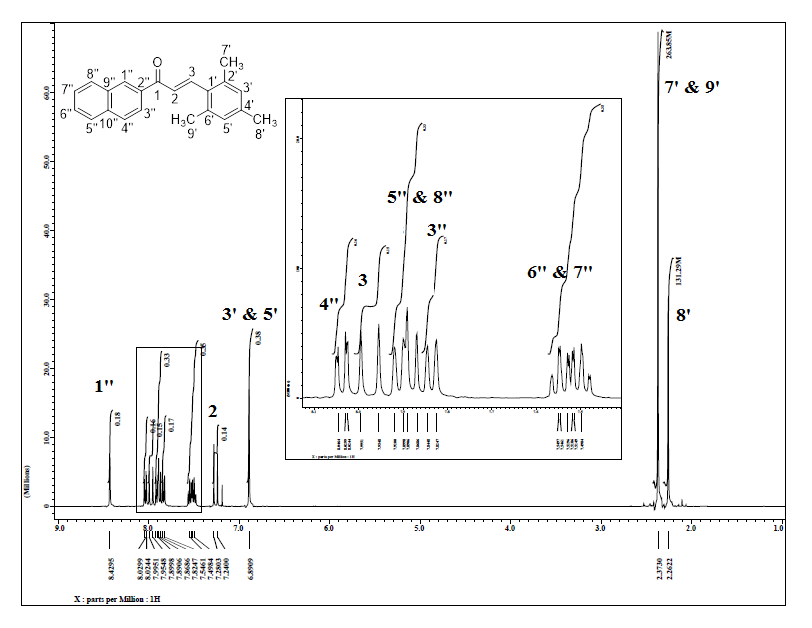
**

**Fig. S1** ^1^H-NMR of the synthesized compound **3**

^
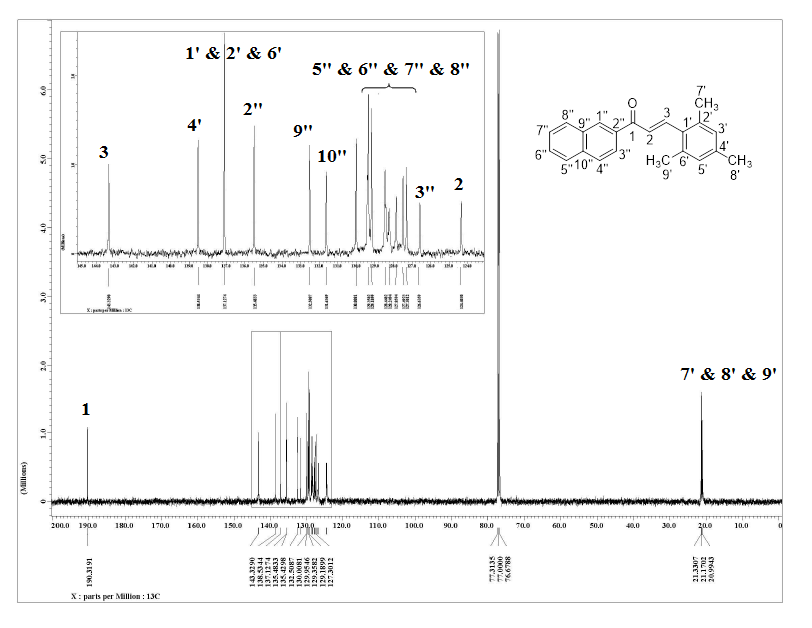
^

**Fig. S2** ^13^C-NMR of the synthesized compound **3**

| **Table S1.** [Geometric parameters (Å, °)](file:///C:\Users\ambarakat\AppData\Local\Temp\Abh47%20_geom_bond_distance)compound **3**  **Bond lengths** |
| --- |

| O1—C11 | 1.228 (2) | C13—C14 | 1.469 (2) |
| --- | --- | --- | --- |
| C1—C2 | 1.365 (2) | C13—H13A | 0.9300 |
| C1—C10 | 1.421 (2) | C14—C15 | 1.415 (2) |
| C1—H1A | 0.9300 | C14—C19 | 1.416 (2) |
| C2—C3 | 1.425 (2) | C15—C16 | 1.398 (2) |
| C2—H2A | 0.9300 | C15—C20 | 1.510 (2) |
| C3—C4 | 1.418 (2) | C16—C17 | 1.392 (2) |
| C3—C8 | 1.423 (2) | C16—H16A | 0.9300 |
| C4—C5 | 1.371 (3) | C17—C18 | 1.392 (2) |
| C4—H4A | 0.9300 | C17—C21 | 1.508 (2) |
| C5—C6 | 1.414 (3) | C18—C19 | 1.394 (2) |
| C5—H5A | 0.9300 | C18—H18A | 0.9300 |
| C6—C7 | 1.374 (2) | C19—C22 | 1.514 (2) |
| C6—H6A | 0.9300 | C20—H20A | 0.9600 |
| C7—C8 | 1.421 (2) | C20—H20B | 0.9600 |
| C7—H7A | 0.9300 | C20—H20C | 0.9600 |
| C8—C9 | 1.420 (2) | C21—H21A | 0.9600 |
| C9—C10 | 1.378 (2) | C21—H21B | 0.9600 |
| C9—H9A | 0.9300 | C21—H21C | 0.9600 |
| C10—C11 | 1.499 (2) | C22—H22A | 0.9600 |
| C11—C12 | 1.477 (2) | C22—H22B | 0.9600 |
| C12—C13 | 1.342 (2) | C22—H22C | 0.9600 |
| C12—H12A | 0.9300 |  |  |

**Bond angles**

| C2—C1—C10 | 120.67 (16) | C14—C13—H13A | 115.8 |
| --- | --- | --- | --- |
| C2—C1—H1A | 119.7 | C15—C14—C19 | 119.30 (14) |
| C10—C1—H1A | 119.7 | C15—C14—C13 | 123.59 (14) |
| C1—C2—C3 | 120.46 (15) | C19—C14—C13 | 117.08 (14) |
| C1—C2—H2A | 119.8 | C16—C15—C14 | 118.97 (15) |
| C3—C2—H2A | 119.8 | C16—C15—C20 | 117.16 (14) |
| C4—C3—C8 | 119.07 (15) | C14—C15—C20 | 123.83 (14) |
| C4—C3—C2 | 121.73 (15) | C17—C16—C15 | 122.00 (15) |
| C8—C3—C2 | 119.18 (15) | C17—C16—H16A | 119.0 |
| C5—C4—C3 | 120.72 (16) | C15—C16—H16A | 119.0 |
| C5—C4—H4A | 119.6 | C16—C17—C18 | 118.54 (15) |
| C3—C4—H4A | 119.6 | C16—C17—C21 | 120.59 (16) |
| C4—C5—C6 | 120.31 (16) | C18—C17—C21 | 120.87 (16) |
| C4—C5—H5A | 119.8 | C17—C18—C19 | 121.46 (16) |
| C6—C5—H5A | 119.8 | C17—C18—H18A | 119.3 |
| C7—C6—C5 | 120.41 (16) | C19—C18—H18A | 119.3 |
| C7—C6—H6A | 119.8 | C18—C19—C14 | 119.67 (15) |
| C5—C6—H6A | 119.8 | C18—C19—C22 | 118.52 (15) |
| C6—C7—C8 | 120.51 (15) | C14—C19—C22 | 121.74 (15) |
| C6—C7—H7A | 119.7 | C15—C20—H20A | 109.5 |
| C8—C7—H7A | 119.7 | C15—C20—H20B | 109.5 |
| C9—C8—C7 | 122.09 (14) | H20A—C20—H20B | 109.5 |
| C9—C8—C3 | 118.92 (15) | C15—C20—H20C | 109.5 |
| C7—C8—C3 | 118.96 (15) | H20A—C20—H20C | 109.5 |
| C10—C9—C8 | 120.71 (15) | H20B—C20—H20C | 109.5 |
| C10—C9—H9A | 119.6 | C17—C21—H21A | 109.5 |
| C8—C9—H9A | 119.6 | C17—C21—H21B | 109.5 |
| C9—C10—C1 | 119.95 (15) | H21A—C21—H21B | 109.5 |
| C9—C10—C11 | 122.56 (15) | C17—C21—H21C | 109.5 |
| C1—C10—C11 | 117.24 (15) | H21A—C21—H21C | 109.5 |
| O1—C11—C12 | 119.81 (15) | H21B—C21—H21C | 109.5 |
| O1—C11—C10 | 119.15 (15) | C19—C22—H22A | 109.5 |
| C12—C11—C10 | 121.04 (14) | C19—C22—H22B | 109.5 |
| C13—C12—C11 | 123.44 (15) | H22A—C22—H22B | 109.5 |
| C13—C12—H12A | 118.3 | C19—C22—H22C | 109.5 |
| C11—C12—H12A | 118.3 | H22A—C22—H22C | 109.5 |
| C12—C13—C14 | 128.41 (15) | H22B—C22—H22C | 109.5 |
| C12—C13—H13A | 115.8 |  |  |

**Torsion angles**

| C10—C1—C2—C3 | 1.1 (3) | C1—C10—C11—C12 | −143.56 (16) |
| --- | --- | --- | --- |
| C1—C2—C3—C4 | 175.56 (17) | O1—C11—C12—C13 | −160.09 (18) |
| C1—C2—C3—C8 | −3.2 (3) | C10—C11—C12—C13 | 19.8 (3) |
| C8—C3—C4—C5 | −0.1 (3) | C11—C12—C13—C14 | 177.86 (16) |
| C2—C3—C4—C5 | −178.87 (17) | C12—C13—C14—C15 | −31.9 (3) |
| C3—C4—C5—C6 | −0.7 (3) | C12—C13—C14—C19 | 150.16 (19) |
| C4—C5—C6—C7 | 0.2 (3) | C19—C14—C15—C16 | −2.3 (2) |
| C5—C6—C7—C8 | 1.2 (3) | C13—C14—C15—C16 | 179.84 (16) |
| C6—C7—C8—C9 | 176.00 (17) | C19—C14—C15—C20 | 175.20 (16) |
| C6—C7—C8—C3 | −2.0 (3) | C13—C14—C15—C20 | −2.6 (3) |
| C4—C3—C8—C9 | −176.59 (16) | C14—C15—C16—C17 | 0.9 (3) |
| C2—C3—C8—C9 | 2.2 (2) | C20—C15—C16—C17 | −176.80 (17) |
| C4—C3—C8—C7 | 1.4 (2) | C15—C16—C17—C18 | 1.5 (3) |
| C2—C3—C8—C7 | −179.80 (16) | C15—C16—C17—C21 | −178.14 (17) |
| C7—C8—C9—C10 | −177.13 (16) | C16—C17—C18—C19 | −2.5 (3) |
| C3—C8—C9—C10 | 0.8 (2) | C21—C17—C18—C19 | 177.15 (17) |
| C8—C9—C10—C1 | −2.9 (2) | C17—C18—C19—C14 | 1.1 (3) |
| C8—C9—C10—C11 | 171.19 (15) | C17—C18—C19—C22 | −175.73 (16) |
| C2—C1—C10—C9 | 1.9 (3) | C15—C14—C19—C18 | 1.4 (2) |
| C2—C1—C10—C11 | −172.48 (16) | C13—C14—C19—C18 | 179.35 (16) |
| C9—C10—C11—O1 | −137.88 (19) | C15—C14—C19—C22 | 178.06 (16) |
| C1—C10—C11—O1 | 36.4 (2) | C13—C14—C19—C22 | −4.0 (2) |
| C9—C10—C11—C12 | 42.2 (2) |  |  |

**Table S2.** The calculated electronic transitions using TD-DFT method.

| λ_max_(nm) | f | Major contributions |
| --- | --- | --- |
| 356.6 | 0.0211 | H-4→L (23%), H-3→L (44%), H-1→L (10%), H→L (11%) |
| 338.0 | 0.1023 | H→L (79%) |
| 317.7 | 0.2765 | H-3→L (23%), H-1→L (63%) |
| 301.4 | 0.0099 | H-2→L (89%) |
| 286.6 | 0.0340 | H-4→L (34%), H-1→L+1 (25%) |
| 283.8 | 0.2028 | H-1→L+1 (12%), H→L+1 (69%) |
| 278.2 | 0.0199 | H-4→L (15%), H-1→L+1 (48%), H→L+2 (14%) |
| 270.2 | 0.0373 | H-4→L (11%), H-3→L+1 (59%), H→L+2 (14%) |
| 258.0 | 0.0008 | H-2→L+1 (75%) |
| 257.3 | 0.0261 | H-4→L+1 (57%), H-3→L+1 (10%) |
| 246.1 | 0.0631 | H-1→L+2 (69%), H→L+2 (15%) |
| 234.7 | 0.0144 | H-2→L+1 (12%), H-2→L+2 (34%), H-1→L+3 (25%), H→L+3 (16%) |
| 232.3 | 0.0064 | H-4→L+2 (20%), H-3→L+2 (62%) |
| 228.5 | 0.0684 | H-6→L (20%), H-5→L (50%) |
| 225.2 | 0.2204 | H-6→L (62%), H-5→L (10%) |
| 222.4 | 0.0002 | H-1→L+3 (22%), H→L+3 (72%) |
| 221.0 | 0.2212 | H-5→L (15%), H→L+2 (13%), H→L+4 (31%) |
| 218.3 | 0.0899 | H-2→L+2 (51%), H-1→L+3 (26%) |
| 215.4 | 0.3187 | H-5→L+1 (20%), H-4→L+2 (14%), H→L+4 (11%) |
| 211.2 | 0.0269 | H-2→L+3 (11%), H-1→L+4 (47%) |

**Table S3**. The calculated chemical shifts δ(ppm) of the studied compound using GIAO method.

| Atom | δ_calc_ (ppm) | δ_exp._(ppm) | Atom | δ_calc_ (ppm) | δ_exp._(ppm) |
| --- | --- | --- | --- | --- | --- |
| C2 | 133.33 | 126.6 | H3 | 8.31 | 8.04 |
| C4 | 134.12 | 127.5 | H5 | 8.11 | 7.84 |
| C6 | 141.31 | 132.5 | H8 | 8.06 | 7.92 |
| C7 | 133.47 | 129.4 | H10 | 7.76 | 7.54 |
| C9 | 133.02 | 127.9 | H12 | 7.70 | 7.49 |
| C11 | 131.53 | 127.3 | H14 | 8.09 | 7.89 |
| C13 | 135.03 | 128.4 | H17 | 8.50 | 8.43 |
| C15 | 138.11 | 131.6 | H21 | 6.73 | 7.28 |
| C16 | 136.11 | 130.0 | H23 | 7.64 | 7.99 |
| C18 | 141.88 | 137.1 | H27 | 7.16 | 6.89 |
| C19 | 199.45 | 190.3 | H30 | 7.12 | 6.89 |
| C20 | 139.27 | 124.5 | H33 | 2.23 | 2.37 |
| C22 | 149.91 | 143.3 | H34 | 2.79 | 2.37 |
| C24 | 138.27 | 137.1 | H35 | 2.66 | 2.37 |
| C25 | 145.59 | 137.1 | H37 | 2.00 | 2.26 |
| C26 | 136.09 | 129.3 | H38 | 2.32 | 2.26 |
| C28 | 146.04 | 138.5 | H39 | 2.55 | 2.26 |
| C29 | 134.01 | 129.4 | H41 | 2.10 | 2.37 |
| C31 | 145.77 | 137.1 | H42 | 2.43 | 2.37 |
| C32 | 25.68 | 21.2 | H43 | 2.61 | 2.37 |
| C36 | 23.48 | 21.3 |  |  |  |
| C40 | 24.78 | 21.0 |  |  |  |

**Table S4** The calculated unscaled and scaled infrared vibrational frequencies of the studied compound.

| **(υ_calc_)_unscal_** | **(υ_calc_)_scal_^a^** | **υ_exp_** | **Intensity (km/mol)** |
| --- | --- | --- | --- |
| 3202 | 3096 |  | 5.314 |
| 3197 | 3092 | 3090 | 7.648 |
| 3189 | 3084 |  | 24.818 |
| 3184 | 3079 |  | 7.236 |
| 3177 | 3072 |  | 27.816 |
| 3166 | 3061 | 3053 | 7.987 |
| 3163 | 3059 |  | 4.024 |
| 3159 | 3055 |  | 2.181 |
| 3157 | 3053 |  | 7.547 |
| 3153 | 3048 | 3030 | 24.813 |
| 3150 | 3046 |  | 25.548 |
| 3113 | 3011 | 3016 | 13.207 |
| 3107 | 3005 | 3007 | 20.584 |
| 3104 | 3002 | 2977 | 16.288 |
| 3083 | 2981 | 2955 | 22.129 |
| 3074 | 2973 | 2919 | 21.047 |
| 3072 | 2971 |  | 15.286 |
| 3028 | 2928 |  | 15.683 |
| 3024 | 2924 | 2852 | 21.581 |
| 3022 | 2922 |  | 38.962 |
| 1706 | 1649 | 1626 | 276.412 |
| 1669 | 1614 | 1604 | 49.542 |
| 1666 | 1611 |  | 32.925 |
| 1647 | 1592 |  | 95.423 |
| 1641 | 1587 |  | 8.171 |
| 1609 | 1555 | 1546 | 2.292 |
| 1598 | 1545 | 1510 | 19.785 |
| 1540 | 1489 | 1500 | 0.355 |
| 1516 | 1466 | 1469 | 16.057 |
| 1504 | 1455 | 1457 | 39.484 |
| 1498 | 1448 | 1442 | 16.103 |
| 1497 | 1447 |  | 6.734 |
| 1493 | 1444 |  | 3.064 |
| 1492 | 1443 |  | 11.158 |
| 1490 | 1441 | 1433 | 8.072 |
| 1467 | 1419 |  | 1.949 |
| 1465 | 1416 |  | 7.368 |
| 1443 | 1396 | 1391 | 2.145 |
| 1419 | 1373 |  | 1.473 |
| 1417 | 1370 |  | 2.469 |
| 1414 | 1367 | 1365 | 1.039 |
| 1408 | 1362 | 1353 | 11.440 |
| 1395 | 1349 | 1345 | 4.984 |
| 1379 | 1333 |  | 14.983 |
| 1363 | 1318 | 1299 | 3.672 |
| 1322 | 1278 | 1290 | 2.401 |
| 1308 | 1265 |  | 207.167 |
| 1305 | 1262 |  | 49.363 |
| 1291 | 1249 |  | 31.104 |
| 1288 | 1245 | 1237 | 5.486 |
| 1270 | 1228 |  | 0.825 |
| 1257 | 1215 |  | 81.215 |
| 1229 | 1189 | 1193 | 0.709 |
| 1215 | 1175 |  | 89.297 |
| 1179 | 1140 | 1145 | 7.013 |
| 1173 | 1134 | 1138 | 3.822 |
| 1172 | 1133 | 1130 | 0.977 |
| 1154 | 1116 |  | 45.955 |
| 1084 | 1049 | 1070 | 30.609 |
| 1060 | 1025 | 1030 | 8.031 |
| 1058 | 1023 |  | 2.655 |
| 1056 | 1021 |  | 3.761 |
| 1048 | 1014 | 1015 | 9.333 |
| 1042 | 1008 |  | 4.452 |
| 1035 | 1001 |  | 5.212 |
| 1034 | 1000 |  | 1.675 |
| 1029 | 995 | 996 | 27.436 |
| 1000 | 967 | 975 | 0.136 |
| 993 | 960 | 960 | 2.333 |
| 979 | 947 | 945 | 0.713 |
| 969 | 937 |  | 3.731 |
| 965 | 933 | 930 | 3.153 |
| 949 | 918 | 916 | 4.211 |
| 935 | 904 |  | 7.715 |
| 913 | 883 | 885 | 31.839 |
| 899 | 870 | 870 | 0.063 |
| 884 | 855 | 853 | 10.004 |
| 877 | 848 |  | 4.267 |
| 870 | 841 |  | 24.781 |
| 844 | 816 | 827 | 24.234 |
| 822 | 795 |  | 3.459 |
| 791 | 765 | 760 | 11.220 |
| 781 | 755 |  | 3.427 |
| 774 | 748 | 745 | 44.890 |
| 754 | 729 | 699 | 2.760 |
| 715 | 691 | 680 | 12.156 |
| 696 | 673 | 640 | 19.012 |
| 650 | 629 | 623 | 0.473 |
| 640 | 619 |  | 4.147 |
| 608 | 588 | 593 | 10.212 |
| 582 | 562 | 555 | 3.851 |
| 572 | 553 | 540 | 0.640 |
| 568 | 549 | 531 | 3.506 |
| 537 | 520 |  | 9.509 |
| 529 | 511 |  | 5.943 |
| 525 | 508 |  | 1.090 |
| 524 | 506 |  | 0.086 |
| 500 | 483 | 488 | 9.664 |
| 489 | 473 |  | 13.180 |
| 461 | 446 |  | 0.919 |
| 404 | 390 |  | 1.135 |
| 396 | 383 |  | 1.520 |
| 367 | 355 |  | 6.327 |
| 351 | 339 |  | 2.278 |
| 334 | 323 |  | 1.552 |
| 308 | 298 |  | 1.105 |
| 283 | 273 |  | 0.377 |
| 246 | 238 |  | 3.069 |
| 232 | 224 |  | 1.960 |
| 222 | 215 |  | 0.486 |
| 196 | 190 |  | 1.970 |
| 191 | 185 |  | 0.744 |
| 184 | 178 |  | 2.473 |
| 167 | 161 |  | 3.968 |
| 161 | 155 |  | 9.539 |
| 138 | 133 |  | 6.389 |
| 118 | 115 |  | 1.005 |
| 83 | 80 |  | 1.983 |
| 55 | 54 |  | 0.658 |
| 48 | 47 |  | 0.253 |
| 35 | 33 |  | 0.071 |
| 28 | 27 |  | 0.588 |
| 16 | 15 |  | 0.285 |

**^a^ Reference [1]**

**Table S5:** PASS prediction of the compound, **Pa** represents probability to be active and **Pi** represents probability to be inactive.

| **Pa** | **Pi** | **Predicted activity** |
| --- | --- | --- |
| 0.391 | 0.052 | Chenodeoxycholoyltaurine hydrolase inhibitor |
| 0.350 | 0.012 | CYP1A1 inhibitor |
| 0.371 | 0.036 | CYP1A inhibitor |
| 0.340 | 0.006 | Skin whitener |
| 0.395 | 0.062 | CYP2A8 substrate |
| 0.383 | 0.050 | Sigma receptor agonist |
| 0.347 | 0.015 | CTGF expression inhibitor |
| 0.432 | 0.100 | CYP2C8 inhibitor |
| 0.420 | 0.088 | Preneoplastic conditions treatment |
| **0.348** | 0.018 | **Histone acetyltransferase inhibitor** |
| 0.346 | 0.017 | CYP2A6 inhibitor |
| 0.399 | 0.072 | APOA1 expression enhancer |
| 0.390 | 0.063 | Centromere associated protein inhibitor |
| 0.441 | 0.114 | Taurine dehydrogenase inhibitor |
| 0.380 | 0.056 | Reductant |
| 0.382 | 0.059 | Cyclohexanone monooxygenase inhibitor |
| 0.375 | 0.052 | N-formylmethionyl-peptidase inhibitor |
| 0.381 | 0.058 | CYP2C18 substrate |
| 0.328 | 0.006 | Lipoxygenase inhibitor |
| 0.354 | 0.033 | Uroporphyrinogen-III synthase inhibitor |

[1]S. P. V. Chamundeeswari, E. R. J. J. Samuel, N. SundaraganesanEurop. J. Chem. 2 (2) (2011) 136‐145.
